# Completion rates and myelosuppression degrees of cancer patients receiving radiotherapy or chemoradiotherapy unchanged regardless of delay duration after Omicron infection

**DOI:** 10.1038/s41598-024-65019-y

**Published:** 2024-06-20

**Authors:** Zhenyu Zhang, Juan Zhou, Xun Peng, Ping Li, Xue Meng, Man Hu, Miaoqing Zhao, Qinghai Lin, Kun Ru

**Affiliations:** 1 Department of Pathology and Lab Medicine, Shandong Cancer Hospital and Institute, Shandong First Medical University and Shandong Academy of Medical Sciences, 440 Jiyan Road, Jinan, 250117 Shandong China; 2 Department of Hematology and Oncology, Shandong Cancer Hospital and Institute, Shandong First Medical University and Shandong Academy of Medical Sciences, Jinan, 250117 China; 3 Department of Radiation Oncology, Shandong Cancer Hospital and Institute, Shandong First Medical University and Shandong Academy of Medical Sciences, Jinan, 250117 China

**Keywords:** Omicron, Cancer patients, Radiotherapy, Chemoradiotherapy, Completion rates, Myelosuppression, Cancer, Diseases, Infectious diseases

## Abstract

This study aimed to investigate impacts of Omicron infection on cancer patients in China. A retrospective study was conducted, including 347 cancer patients undergoing radiotherapy or chemoradiotherapy between July 2022 and March 2023. Three groups involved: 108 patients without SARS-CoV-2 infection (non-COVID-19 group), 102 patients beginning treatment 10 days after first SARS-CoV-2 infection (≥ 10 days COVID-19 group), and 137 patients beginning treatment less than 10 days after first SARS-CoV-2 infection (< 10 days COVID-19 group). SAA, hsCRP, ALT, etc., were used to assess COVID-19 infection. Serum levels of SAA, hsCRP and IL-6 were all raised in two COVID-19-infected groups (SAA < 0.01, hsCRP < 0.01, IL-6 < 0.05), but PCT, ALT, LDH and HBDH levels were only elevated in ≥ 10 days COVID-19 group (PCT = 0.0478, ALT = 0.0022, LDH = 0.0313, HBDH = 0.0077). Moreover, moderate and severe infected cases were higher in ≥ 10 days COVID-19 group than < 10 days COVID-19 group (12/102 vs 5/137, p = 0.0211), but no significance in myelosuppression and completion rates among three groups. Omicron infection led to inflammation, liver and cardiovascular injury on cancer patients, but delay duration of radiotherapy or chemoradiotherapy after infection did not affect the completion rates and myelosuppression of current therapy. Besides, severity of Omicron infection was even worse among cancer patients who received delayed treatment.

## Introduction

According to data from the World Health Organization (WHO), there have been 764,474,387 confirmed cases of the 2019 novel coronavirus disease (COVID-19) till April 24, 2023, with 6,915,286 deaths since the start of the pandemic (WHO, 2023)^1^. In China, 99,244,445 confirmed cases have been reported, with over 40,000,000 cases infected by the Omicron variant by the end of 2022^2^.

Apart from its impact on the respiratory system, COVID-19 can affect various organs and physiological functions, including the inflammatory response, oxidative stress, coagulation, and immunity^3–5^. Previous studies reported that immunocompromised cancer patients are more susceptible to COVID-19 infection, with a higher risk of poor outcomes^6,7^. Cancer patients infected with COVID-19 develop severe symptoms, which can be attributed to both tumor growth and anti-cancer treatment^8,9^. As chemotherapy or radiotherapy may increase the risk of adverse events in patients infected with COVID-19, the National Comprehensive Cancer Network (NCCN) guidelines recommend cautious treatment decisions for cancer patients positive for COVID-19^10–12^. The NCCN guidelines version 3.2022 Prevention and treatment of cancer-related infections recommends that cancer patients with mild to moderate COVID-19 should not undergo radiation therapy, targeted therapy, long-acting biologic therapy, or immunotherapy until at least 10 days after their SARS-CoV-2 infection^13^.

However, some studies revealed that treatment delays caused by the COVID-19 pandemic can shorten the survival of cancer patients^14^. Furthermore, radiotherapy may improve COVID-19 infection^15,16^. These discrepancies suggest that the relationship between COVID-19 and cancer treatment should be re-assessed. Since COVID-19 is no longer considered a public health emergency of international concern, it is worth addressing whether NCCN guidelines should still be considered.

In this retrospective study, we investigated the effect of SARS-CoV-2 Omicron variant infection on cancer patients and cancer treatment, providing clinical evidence for determining the appropriate timing of treatment for cancer patients infected with Omicron.

## Results

### Clinical characteristics of cancer patients

The clinical characteristics of all cancer patients were presented in Table 1. We found a higher proportion of patients with breast cancer in the < 10 days COVID-19 group compared with the other groups (p = 0.0278). Similarly, there were more women (p = 0.0160) and a higher number of cancer patients undergoing breast and chest radiotherapy in the < 10 days COVID-19 group (p = 0.0312). Additionally, the taxol (plant alkaloids)/platinum/cytotoxicity drugs were used less in the < 10 days COVID-19 group than in other groups (p = 0.0185). Since this study included all cancer patients infected with COVID-19 from November 2022 to March 2023, it indicated that clinicians were more conservative when treating cancer patients infected with COVID-19 within the first 10 days.

**Table 1:** The clinical characteristics of all cancer patients.

Clinical characteristics	Non-COVID-19 group (n = 108)	< 10 days COVID-19 group (n = 137)	≥ 10 days COVID-19 group (n = 102)	p value
Age (year)	58.42 ± 12.81	57.86 ± 10.82	58.63 ± 11.09	0.7324
Sex, female (%)	33 (30.56)	65 (47.45)**	35 (34.31)	0.0160
Cancer type, n (%)				0.0578
Lung cancer	46 (42.59)	68 (49.64)	55 (53.92)	
Breast cancer	9 (8.33)	25 (18.25)*	9 (8.82)	0.0278
Esophageal cancer	18 (16.67)	10 (7.30)	11 (10.78)	
Head and neck cancer	17 (15.74)	12 (8.76)	13 (12.75)	
Brain cancer	8 (7.41)	5 (3.65)	6 (5.88)	
Other	10 (9.26)	17 (12.41)	8 (7.84)	
Radiotherapy position, n (%)				0.0175
Lung	26 (24.07)	30 (21.90)	29 (28.43)	
Breast and chest	12 (11.11)	30 (21.90)*	12 (11.76)	0.0312
Head and neck	19 (17.59)	11 (8.03)	12 (11.76)	
Brain	18 (16.67)	29 (21.17)	16 (15.69)	
Bone	13 (12.04)	11 (8.03)	13 (12.75)	
Esophagus and other positions	20 (18.52)	26 (18.98)	20 (19.61)	
Drug, n (%)	N = 72	N = 83	N = 59	0.0142
Taxol (plant alkaloids) /platinum/cytotoxicity	47 (65.28)	37 (44.58)*	37 (62.71)	0.0185
Targeting drugs	16 (22.22)	18 (21.69)	15 (25.42)	
Immunotherapy	1 (1.39)	10 (12.05)	1 (1.69)	
Endocrine therapy	3 (4.17)	11 (13.25)	3 (5.08)	
Other	5 (6.94)	7 (8.43)	3 (5.08)	
Duration of the delaying therapy after COVID-19 (days)	–	3.02 ± 3.23 (0–9)	16.36 ± 6.57 (10–39)	< 0.0001

p value was calculated by Mann–Whitney *U* test between two groups. Comparison among three groups was analyzed by one-way ANOVA test for normally distributed data, or Kruskal–Wallis test for abnormally distributed data. Chi square test and Fisher’s exact test were used for analyzing the rates. The numerical value was represented by mean ± SD.

*p < 0.05 vs. non-COVID-19 group. **p < 0.01 vs. non-COVID-19 group.

Table 2 presented the complete blood count (CBC) results of all cancer patients before treatment. It was observed that the lymphocyte levels in both COVID-19-infected groups were lower than those in patients without COVID-19 infection, which was consistent with the characteristics of COVID-19 infection reported. Moreover, according to the mean values of three groups, lymphocyte counts were inversely proportional to the COVID-19 infection time. Correspondingly, the change in neutrophil to lymphocyte ratio (NLR) exhibited a similar pattern to that of lymphocyte counts, which showed much higher NLR in ≥ 10 days COVID-19 group compared with non-COVID-19 group, but no significant difference was found between < 10 days COVID-19 group and non-COVID-19 group. Nevertheless, the average lymphocyte counts in all three groups were still within the normal range. Other hematological variables, including white blood cell count (WBC), neutrophils (NEU), lymphocyte percentage (LYM%), hemoglobin (HGB), and platelets (PLT), did not demonstrate statistical differences among the three groups.

**Table 2:** The CBC results of all cancer patients before treatment.

Laboratory results	Non-COVID-19 group (n = 108)	< 10 days COVID-19 group (n = 137)	≥ 10 days COVID-19 group (n = 102)	p value
WBC (10^9^/L) (3.5–9.5)	6.42 ± 3.24	5.89 ± 3.44	6.54 ± 3.31	0.1106
NEU (10^9^/L) (1.8–6.3)	4.27 ± 2.97	4.05 ± 3.41	4.53 ± 3.06	0.1353
LYM (10^9^/L) (1.1–3.2)	1.67 ± 1.15	1.41 ± 0.75**	1.35 ± 0.56*	0.0123
LYM% (20–50)	26.72 ± 11.65	26.38 ± 13.15	23.67 ± 10.47	0.1589
HGB (g/L) (115–150)	122.9 ± 19.19	122.9 ± 17.29	121.3 ± 18.46	0.7511
PLT (10^9^/L) (125–350)	228.6 ± 77.82	226.3 ± 87.97	237.2 ± 88.07	0.5997
NLR	2.94 ± 2.13	3.87 ± 4.42	4.42 ± 5.49*	0.0540

p value was calculated by Mann–Whitney *U* test between two groups. Comparison among three groups was analyzed by one-way ANOVA test for normally distributed data, or Kruskal–Wallis test for abnormally distributed data. The numerical value was represented by mean ± SD.

*p < 0.05 vs. non-COVID-19 group. **p < 0.01 vs. non-COVID-19 group.

### Severity of COVID-19-related inflammation among the three groups

**Figure 1:**
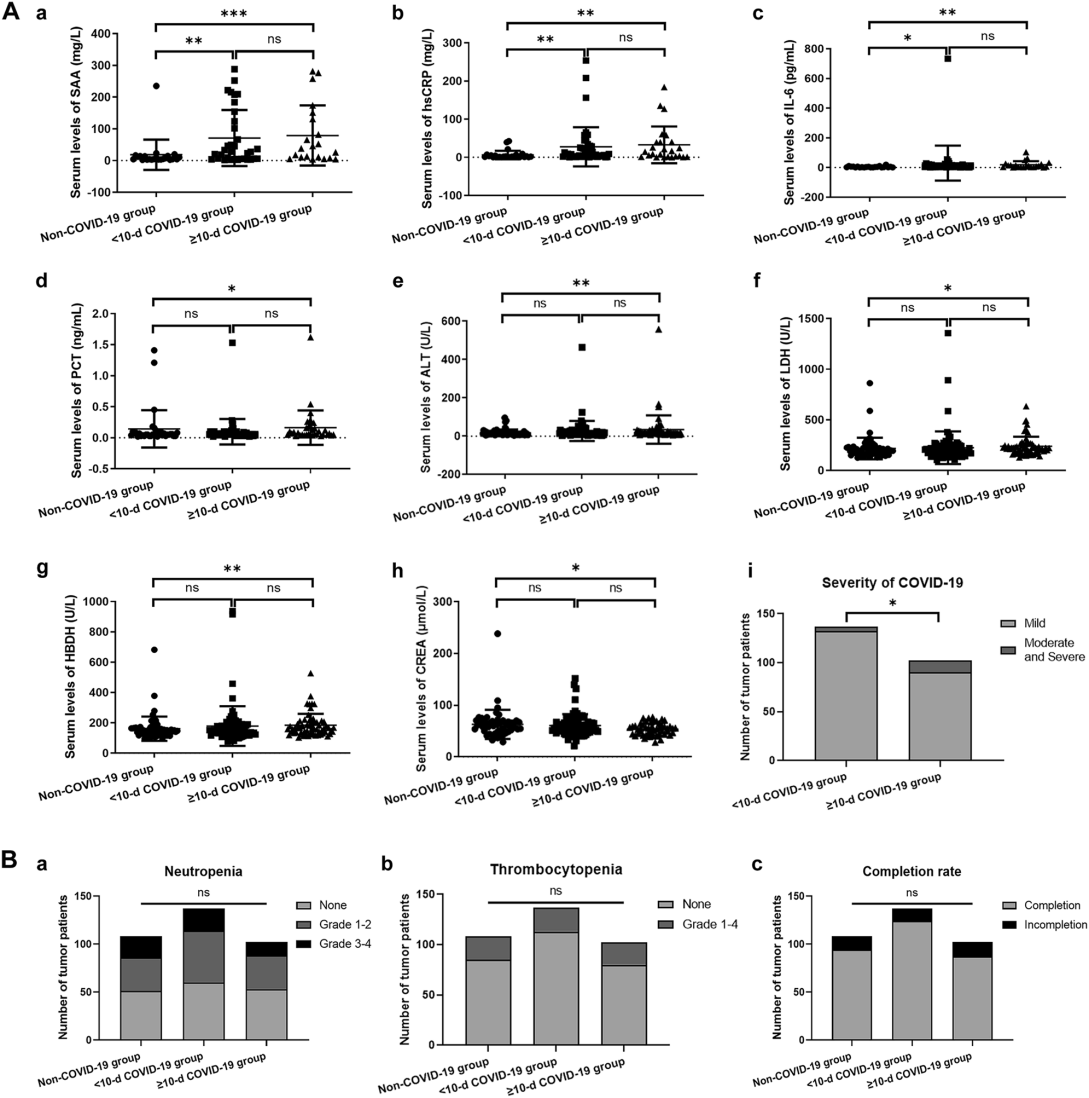
The COVID-19 infection situations, completion rates and myelosuppression degrees of cancer patients involved. (**A**) Omicron related inflammation, liver injury and myocardial injury among three groups. (a), SAA levels; (b), hsCRP levels; (c), IL-6 levels; (d), PCT levels; (e), ALT levels; (f), LDH levels; (g), HBDH levels; (h), CREA levels; (i), severity of COVID-19 in two Omicron infected groups. (**B**) The myelosuppression degrees and completion rates of current therapy period among three groups. (a), Neutropenia degrees; (b), Thrombocytopenia degrees; (c), completion rates of current therapy period. *p < 0.05 vs. non-COVID-19 group. **p < 0.01 vs. non-COVID-19 group. ***p < 0.001 vs. non-COVID-19 group.

Serum levels of serum amyloid protein A (SAA), high-sensitivity C-reactive protein (hsCRP), interleukin-6 (IL-6), procalcitonin (PCT), prealbumin (PA), and ferritin, were measured to assess the effect of Omicron-related inflammation among cancer patients. The results in Table 3 and Fig. 1 demonstrated significantly higher levels of SAA, hsCRP, and IL-6 in the two COVID-19-infected groups compared with the non-COVID-19 group (p < 0.05 for all). Nevertheless, no significant difference was observed between < 10 days COVID-19 group and ≥ 10 days COVID-19 group. Interestingly, the PCT level was notably elevated in ≥ 10 days COVID-19 group compared with non-COVID-19 group (p = 0.0478), whereas there was no significance between < 10 days COVID-19 group and non-COVID-19 group. Consistently, compared to non-COVID-19 group, the average values of SAA, hsCRP and IL-6 in ≥ 10 days COVID-19 group were all higher than that in < 10 days COVID-19 group (Table 3). Furthermore, the ratio of moderate and severe COVID-19 cases was significantly higher in ≥ 10 days COVID-19 group compared with < 10 days COVID-19 group (p = 0.0211). It is worth noting that although physicians typically used CRP to assess the severity of infection, the serum level of SAA in this study was significantly higher than the serum level of CRP.

**Table 3:** Severity of COVID-19 related inflammation among three groups.

Laboratory results (normal range)	Non-COVID-19 group	< 10 days COVID-19 group	≥ 10 days COVID-19 group	p value
SAA (mg/L) (0–10)	18.33 ± 47.65 (n = 23)	71.03 ± 88.35 (n = 34)**	79.13 ± 94.87 (n = 21)***	0.0022
hsCRP (mg/L) (0–10)	6.09 ± 11.18 (n = 26)	27.53 ± 51.37 (n = 47)**	32.73 ± 48.20 (n = 25)**	0.0045
IL-6 (pg/mL) (0–7)	4.65 ± 4.25 (n = 22)	11.26 ± 12.41 (n = 37)*	14.41 ± 16.25 (n = 21)**	0.0225
PCT (ng/mL) (0–0.05)	0.14 ± 0.30 (n = 35)	0.10 ± 0.21 (n = 54)	0.16 ± 0.28 (n = 35)*	0.0792
Ferritin (ng/mL) (30–400)	366.2 ± 383.9 (n = 22)	600.0 ± 607.9 (n = 35)	961.2 ± 1524 (n = 21)	0.1739
PA (g/L) (0.25–0.4)	0.24 ± 0.09 (n = 65)	0.21 ± 0.08 (n = 84)	0.22 ± 0.09 (n = 61)	0.2421
Severity of COVID-19, n (%)				
Mild	–	132 (96.35)	90 (88.24)	0.0211
Moderate and severe	–	5 (3.65)	12 (11.76)	

p value was calculated by Mann–Whitney *U* test between two groups. Comparison among three groups was analyzed by one-way ANOVA test for normally distributed data, or Kruskal–Wallis test for abnormally distributed data. Fisher’s exact test was used for analyzing the rates. The numerical value was represented by mean ± SD.

*p < 0.05 vs. non-COVID-19 group. **p < 0.01 vs. non-COVID-19 group. ***p < 0.001 vs. non-COVID-19 group.

### Comparison of liver function, renal function, and myocardial injury biomarkers

Table 4 and Fig. 1 presented the serum levels of alanine aminotransferase (ALT), aspartate aminotransferase (AST), lactate dehydrogenase (LDH), hydroxybutyrate dehydrogenase (HBDH), creatine kinase (CK), creatine kinase MB (CK-MB), glutamyltransferase (GGT), cholinesterase (CHE), creatinine (CREA), cardiac troponin-T (cTnT), and pro B-type natriuretic peptide (proBNP), which represented the liver function, renal function, and myocardial injury during anti-cancer treatment. Compared with non-COVID-19 group, the numerical values of ALT (p = 0.0022), LDH (p = 0.0313), and HBDH (p = 0.0077) were all significantly elevated in ≥ 10 days COVID-19 group, but no significant differences observed in < 10 days COVID-19 group.

**Table 4:** Biomarkers of liver function, renal function and myocardial injury during treatment.

Laboratory results	Non-COVID-19 group	< 10 days COVID-19 group	≥ 10 days COVID-19 group	p value
ALT (U/L) (7–40)	18.37 ± 16.56 (n = 65)	28.05 ± 52.35 (n = 83)	35.28 ± 73.97 (n = 61) **	0.0142
AST (U/L) (13–35)	19.80 ± 10.88 (n = 65)	23.61 ± 20.31 (n = 84)	27.16 ± 37.62 (n = 61)	0.3404
LDH (U/L) (109–245)	217.0 ± 105.8 (n = 65)	224.7 ± 161.2 (n = 84)	238.7 ± 94.35 (n = 61)*	0.0344
HBDH (U/L) (72–182)	161.2 ± 80.04 (n = 65)	178.6 ± 130.7 (n = 84)	184.4 ± 75.12 (n = 61)**	0.0281
CK (U/L) (26–140)	48.62 ± 43.39 (n = 65)	41.62 ± 28.96 (n = 84)	47.66 ± 34.51 (n = 61)	0.2744
CK-MB (ng/mL) (0–5)	3.02 ± 3.33 (n = 65)	2.55 ± 1.25 (n = 84)	2.38 ± 0.98 (n = 61)	0.4915
GGT (U/L) (7–45)	38.75 ± 30.64 (n = 65)	42.43 ± 42.00 (n = 83)	73.03 ± 123.5 (n = 61)	0.4149
CHE (U/L) (5000–12,000)	6848 ± 2340 (n = 65)	6747 ± 1944 (n = 83)	6625 ± 1769 (n = 61)	0.8259
CREA (μmol/L) (45–84)	63.14 ± 28.15 (n = 56)	60.83 ± 22.20 (n = 75)	53.64 ± 11.72 (n = 52)*	0.0523
cTnT (pg/mL) (0–14)	12.46 ± 13.65 (n = 26)	12.05 ± 20.24 (n = 49)	10.65 ± 5.39 (n = 29)	0.3213
proBNP (pg/mL) (0–125)	146.5 ± 185.1 (n = 25)	159.4 ± 234.0 (n = 49)	153.0 ± 244.2 (n = 28)	0.7421

p value was calculated by Mann–Whitney *U* test between two groups. Comparison among three groups was analyzed by one-way ANOVA test for normally distributed data, or Kruskal–Wallis test for abnormally distributed data. The numerical value was represented by mean ± SD.

*p < 0.05 vs. non-COVID-19 group. **p < 0.01 vs. non-COVID-19 group.

However, although CREA levels were still within the normal range, the values were evidently descended in ≥ 10 days COVID-19 group compared with non-COVID-19 group (p = 0.0167). The decline degrees in CREA levels displayed a similar pattern as lymphocyte counts, and this might suggest that the longer COVID-19 infected, the more undernourished status cancer patients became. These findings supported the notion that the severity of COVID-19 infection was worse in ≥ 10 days COVID-19 group compared with < 10 days COVID-19 group. Besides, no significant differences were found for other biomarkers.

### The completion rates, CBC, and lymphocyte subtypes after treatment

The degree of myelosuppression after anti-tumor therapy was evaluated using CBC, and the lowest values observed during treatment were summarized in Table 5. Interestingly, no significant differences were found in any of the hematologic indices among the three groups. Although the lymphocyte counts were slightly lower in two COVID-19-infected groups than in Non-COVID-19 group before therapy, the anti-tumor treatment effectively eliminated this small difference.

**Table 5:** The completion rate and CBC (lowest values during treatment) after anti-tumor therapy.

Laboratory results	Non-COVID-19 group (n = 108)	< 10 days COVID-19 group (n = 137)	≥ 10 days COVID-19 group (n = 102)	p value
WBC (10^9^/L) (3.5–9.5)	3.22 ± 1.42	3.37 ± 2.03	3.73 ± 2.15	0.2818
NEU (10^9^/L) (1.8–6.3)	1.98 ± 1.11	2.22 ± 1.97	2.50 ± 1.95	0.1297
LYM (10^9^/L) (1.1–3.2)	0.61 ± 0.43	0.66 ± 0.52	0.59 ± 0.33	0.7033
LYM% (20–50)	11.62 ± 7.43	12.82 ± 8.71	11.56 ± 7.78	0.5075
HGB (g/L) (115–150)	111.8 ± 18.43	110.3 ± 17.26	110.4 ± 21.12	0.8029
PLT (10^9^/L) (125–350)	152.9 ± 70.80	152.2 ± 63.19	162.1 ± 74.75	0.6079
NLR	12.32 ± 18.85	11.09 ± 14.63	15.66 ± 31.53	0.4998
Neutropenia, n (%)				
None	51 (47.22)	60 (43.80)	53 (51.96)	0.5404
Grade 1–2	35 (32.41)	54 (39.42)	35 (34.31)	
Grade 3–4	22 (20.37)	23 (16.79)	14 (13.73)	
Thrombocytopenia, n (%)				
None	85 (78.70)	113 (82.48)	80 (78.43)	0.6709
Grade 1–4	23 (21.30)	24 (17.52)	22 (21.57)	
Completion rate, n (%)				
Completion	94 (87.04)	124 (90.51)	87 (85.29)	0.4483
Incompletion	14 (12.96)	13 (9.49)	15 (14.71)	

p value was calculated by Mann–Whitney *U* test between two groups. Comparison among three groups was analyzed by one-way ANOVA test for normally distributed data, or Kruskal–Wallis test for abnormally distributed data. Chi square test was used for analyzing the rates. The numerical value was represented by mean ± SD.

Similarly, the analysis of lymphocyte subtypes among different groups of cancer patients revealed no significant differences (Supplementary Table S1).

The analysis of electronic medical records revealed that there were 14 patients in non-COVID-19 group, 13 patients in < 10 days COVID-19 group, and 15 patients in ≥ 10 days COVID-19 group who did not complete their treatments. The completion rates of current treatment did not significantly differ among the three groups, irrespective of the presence of Omicron infection and the duration of treatment delay (Table 5 and Fig. 1). Additionally, Supplementary Table S2 and Supplementary Table S3 summarize the cancer types, radiotherapy positions, and incompletion reasons of tumor patients who did not complete treatment.

## Discussion

Although COVID-19 is no longer declared a public health emergency of international concern, it continues to pose a significant challenge to global health^3^. Since late 2022, the Omicron variant has become the dominant strain of SARS-CoV-2 worldwide^17,18^. However, the effects of Omicron infection on patients with cancer are not yet fully understood. In this study, we demonstrated that neither the Omicron infection nor the duration of treatment delay significantly affected the myelosuppression degrees and treatment completion rates of patients with cancer receiving radiotherapy or chemoradiotherapy.

Previous studies have shown that COVID-19 increases the risk of developing severe symptoms among cancer patients, which can be attributed to both tumor growth and anticancer treatment^8,9^. A study conducted in the United States with 507,307 patients with COVID-19 found that patients with cancer are at a greater risk of adverse outcomes, and those undergoing cancer treatment, particularly those with metastasis, suffer from a higher risk of death and hospitalization^19^. Another study reported a significantly higher mortality rate among cancer patients with COVID-19 compared with non-cancer patients who were infected^20^. Therefore, the National Comprehensive Cancer Network (NCCN) recommended delaying anti-tumor treatment for at least 10 days after the first positive test for SARS-CoV-2^13^.

In this study, we analyzed data from three groups of cancer patients: a non-COVID-19 group, a < 10 days COVID-19 group, and a ≥ 10 days COVID-19 group. Omicron infection did influence some inflammatory, liver function, and cardiovascular injury biomarkers, but no significant differences were found in the degree of bone marrow suppression or treatment completion rates, regardless of the duration of treatment delay. It was important to note that, while selection bias related to breast cancer patients was identified in Table 1, the conclusion remained unchanged in the sensitivity analysis after excluding data from breast cancer patients (data not shown). These findings suggest that it is not necessary to delay anti-tumor treatment for cancer patients after Omicron infection, which is not consistent with the recommendations of the NCCN guidelines.

On the other hand, some studies indicated that chemotherapy administered four weeks before COVID-19 infection does not influence the mortality of cancer patients, suggesting that the effects of COVID-19 on cancer treatment may differ at different time points^21^. Additionally, treatment delays caused by the COVID-19 pandemic worsened the survival of cancer patients^14^. Furthermore, several studies suggested that radiotherapy may improve COVID-19 infection^15,16^. Similarly, the results of our study revealed that cancer patients who received delayed radiotherapy or chemoradiotherapy after their COVID-19 infection experienced more severe infection. One possible explanation for this observation is that following the NCCN guidelines, most patients in the ≥ 10 days COVID-19 group were discharged after Omicron infection and returned to the hospital 10 days later. However, early medical interventions alleviated COVID-19 related infection degrees in the < 10 d COVID-19 group, evidenced by the laboratory results, emphasizing the importance of maintaining medical care continuity for all patients to maximize benefits. Although these findings are not sufficient to conclude that radiotherapy can improve COVID-19, they suggest that radiotherapy administered during the early stages of viral infection does not worsen the severity of COVID-19.

Previous studies also indicated that different variants of SARS-CoV-2 can have varying effects on the symptoms and physiological functions of patients. For example, it has been reported that only one-third of patients with hematological neoplasms had antibodies against the delta variant after receiving two doses of vaccine^22^. Another study found that the response of cancer patients to COVID-19 vaccines gradually decreased for the alpha (B.1.1.7), beta (B.1.351), and delta (B.1.617.2) variants, but the immune response increased with a third vaccine dose of for the Omicron variant ^23^. Additionally, a study found significantly lower neutralizing antibody titers for the Omicron variant (B.1.1.529) than for the delta (B.1.617.2) and beta (B.1.351) variants^24^. A recent study from the US reported a mortality rate of 4.9% among cancer patients infected with the Omicron variant, which was significantly lower than that observed among patients infected with the original variant^25^. However, there are few studies on how Omicron or other variants affect cancer patients undergoing radiotherapy or chemoradiotherapy.

Our study observed that the SARS-CoV-2 Omicron variant did not affect treatment completion rates in the current treatment, and the severity of Omicron infection was even worse in cancer patients who received delayed treatment. These results indicate that delayed radiotherapy or chemoradiotherapy is not suitable for cancer patients infected with the Omicron variant. However, this conclusion is based on the immediate effects during the treatment period, and drawing broader conclusions regarding the overall effect of the Omicron variant on cancer patients and cancer treatment may need additional studies.

## Conclusions

In conclusion, we demonstrated that the SARS-CoV-2 Omicron variant promoted the inflammatory response in cancer patients, but the duration of delayed treatment after infection did not affect myelosuppression and the completion rate of current treatment period. This study illuminates the effect of SARS-CoV-2 Omicron infection on cancer treatment and indicates that the therapeutic strategies of cancer patients need some changes in the post-COVID-19 era.

## Methods and materials

### Patients

This retrospective study included 347 adult cancer patients who received radiotherapy or chemoradiotherapy at Shandong Cancer Hospital in China between July 2022 and March 2023. We excluded cancer patients who underwent simple chemotherapy and all underage cancer patients. The primary endpoint was discharge from the hospital based on the medical records. Based on their Omicron infection status and duration of treatment delay after the infection, patients were divided into three groups: a non-COVID-19 group (n = 108), a < 10 days COVID-19 group (n = 137), and a ≥ 10 days COVID-19 group (n 102). The non-COVID-19 group consisted of 108 patients negative for COVID-19 throughout the entire hospitalization period, confirmed by daily PCR tests. This group included 36 patients receiving simple radiotherapy and 72 patients receiving chemoradiotherapy. The < 10 days COVID-19 group included 137 patients who received treatment within 10 days after their first positive COVID-19 test and who received treatment before COVID-19 infection and did not quit the treatment after the infection. In this group, 54 patients received simple radiotherapy, and 83 patients received chemoradiotherapy. The ≥ 10 days COVID-19 group consisted of 102 patients whose radiotherapy or chemoradiotherapy was initiated between 10 and 40 days after the first positive COVID-19 test. This group included 43 patients receiving simple radiotherapy and 59 patients receiving chemoradiotherapy. Patients who received anti-cancer therapy after 40 days were excluded. The duration of treatment delay was assessed from the time of the first positive COVID-19 nucleic acid or antigen test to the start of treatment. We included all COVID-19 cases reported in Shandong Cancer Hospital during the study period. The COVID-19 variants in this study were Omicron strains, which were detected by the Shandong Provincial Center for Disease Control and Prevention. COVID-19 infection was diagnosed based on the quick advice guide for the diagnosis and treatment of COVID-19 provided by the Chinese National Health Commission^26^. The specific protocols used for treating cancer patients based on their individual conditions and chemotherapy agents were summarized in Table 1. Informed consents were obtained from all patients, and the study was approved by the Ethics Committee of Shandong Cancer Hospital.

### Data collection

Three biochemical analyzers were utilized for measuring various biomarkers. The Beckman AU5800 automatic biochemical analyzer (Beckman Coulter, CA, USA) was used to measure inflammatory cytokines, such as serum amyloid protein A (SAA), high-sensitivity C-reactive protein (hsCRP), prealbumin (PA), cardiovascular injury-related biomarkers, including aspartate aminotransferase (AST), serum lactate dehydrogenase (LDH), hydroxybutyrate dehydrogenase (HBDH), creatine kinase (CK), and creatine kinase MB (CK-MB), and liver function biomarkers, including alanine aminotransferase (ALT), glutamyltransferase (GGT), cholinesterase (CHE), and renal function biomarker creatinine (CREA). The Cobas e801 analyzer (Roche Diagnostics, GmbH, Mannheim, Germany) was used to measure the serum levels of ferritin, interleukin-6 (IL-6), procalcitonin (PCT), cardiac troponin-T (cTnT), and pro B-type natriuretic peptide (proBNP). Complete blood count (CBC) was assessed using a Sysmex XN9000 (Sysmex Corporation, Kobe, Japan) to measure white blood cell count (WBC), neutrophils (NEU), lymphocytes (LYM), lymphocyte percentage (LYM%), hemoglobin (HGB), platelets (PLT), and neutrophil to lymphocyte ratio (NLR).

Lymphocyte subtypes were determined using flow cytometry with a NAVIOS flow cytometer (Beckman Coulter, USA). The analyzed lymphocyte subtypes included NK cell (CD3-CD16+CD56+), B cell (CD3-CD19+), total T cell (CD3+), Th cell (CD3+CD4+), Tc/Ts cell (CD3+CD8+), and the ratio of CD4+/CD8+, CD3 +CD4+/CD3+, CD3+CD4+CD25+FOXP3+/CD3+, and CD3+CD4+CD25+FOXP3+/CD3+CD4+. Anti-CD3, anti-CD16, anti-CD56, anti-CD19, anti-CD4, anti-CD8, and anti-CD25 antibodies were used for flow cytometry (Tongsheng Shidai Biotech Co., Ltd, Beijing, China). The anti-FOXP3 antibody was obtained from MultiSciences Biotech Co., Ltd (Hangzhou, Zhejiang, China). All laboratory data were collected following the standard operating procedures in the clinical laboratory of Shandong Cancer Hospital.

Demographic and clinical data, including age, sex, cancer types, position of radiotherapy, duration of therapy delay, as well as laboratory results, were collected from the electronic medical record and laboratory information system (Ruimei LIS version 6.0, Shanghai, China).

### Statistical analysis

Statistical analysis was performed using GraphPad Prism version 9.0 (GraphPad Software, CA, USA). The normality of all data was measured. Data are presented as mean ± SD. The Mann–Whitney *U* test was used to compare two groups with non-normal distribution. The Kruskal–Wallis test was utilized to compare three groups with non-normal distribution, while the one-way analysis of variance (ANOVA) test was employed for normally distributed data. The Chi-square test and Fisher’s exact test were utilized to compare rates. A p-value less than 0.05 was considered statistically significant.

### Ethics approval and consent to participate

This study was approved by Ethics Committee of the Shandong Cancer Hospital and Institute, and conducted in accordance with the local legislation and institutional requirements. The participants provided their written informed consent to participate in this study.

### Supplementary Information

Supplementary Table S1.

Supplementary Table S2.

Supplementary Table S3.

## Data Availability

The data generated in the present study may be requested from the corresponding author.

## Abbreviations

NCCN: National comprehensive cancer network
CBC: Complete blood count
WBC: White blood cell count
NEU: Neutrophils
LYM: Lymphocytes
LYM%: Lymphocyte percentage
HGB: Hemoglobin
PLT: Platelets
NLR: Neutrophil to lymphocyte ratio
SAA: Serum amyloid protein A
hsCRP: High-sensitivity C-reactive protein
IL-6: Interleukin-6
PCT: Procalcitonin
PA: Prealbumin
ALT: Alanine aminotransferase
AST: Aspartate aminotransferase
LDH: Lactate dehydrogenase
HBDH: Hydroxybutyrate dehydrogenase
CK: Creatine kinase
CK-MB: Creatine kinase MB
GGT: Glutamyltransferase
CHE: Cholinesterase
CREA: Creatinine
cTnT: Cardiac troponin-T
proBNP: Pro B-type natriuretic peptide
