# Supplementary material for: Completion rates and myelosuppression degrees of cancer patients receiving radiotherapy or chemoradiotherapy unchanged regardless of delay duration after Omicron infection
Source: Sci Rep. 2024 Jun 20;14:14226. doi: 10.1038/s41598-024-65019-y (PMC11190149; doi:10.1038/s41598-024-65019-y)
Supplement: Supplementary file 1 — Supplementary Table S1. [file 41598_2024_65019_MOESM1_ESM.pdf]

Supplementary Table S1. Comparison of lymphocyte subtypes among three groups

Laboratory results	Non-COVID-19 group	<10-d COVID-19 group	≥10-d COVID-19 group	p value*
NK cell (%) (7-40)	17.84±7.90 (n=15)	18.22±9.48 (n=35)	15.65±7.57 (n=23)	0.5257
B cell (%) (5-18)	7.97±6.14 (n=15)	5.72±5.76 (n=35)	5.96±4.70 (n=23)	0.3951
Total T cell (%) (50-84)	66.83±8.69 (n=15)	67.02±11.15 (n=35)	68.99±11.47 (n=23)	0.7576
Th cell (%) (27-51)	37.21±10.82 (n=15)	36.03±12.97 (n=35)	37.72±14.29 (n=23)	0.8826
Tc/Ts cell (%) (15-44)	21.26±6.46 (n=15)	24.40±10.54 (n=35)	24.76±8.30 (n=23)	0.4488
$CD3^+CD4^+CD25^+$ $FOXP3^+/CD3^+$ (%)	0.99±0.68 (n=18)	1.01±1.05 (n=36)	0.80±0.98 (n=25)	0.4927
$CD3^+CD4^+CD25^+$ $FOXP3^+/CD3^+CD4^+$ (%)	2.12±1.48 (n=18)	1.74±1.53 (n=36)	1.62±1.66 (n=25)	0.3747
$CD3^+CD4^+/CD3^+$ (%)	51.08±12.92 (n=18)	56.12±14.80 (n=36)	53.53±16.19 (n=25)	0.4895
$CD4^+/CD8^+$	1.89±0.79 (n=15)	1.89±1.32 (n=35)	1.84±1.37 (n=23)	0.5003

*p value was calculated by Mann-Whitney U test between two groups. Comparison among three groups was analyzed by one-way ANOVA test for normally distributed data, or Kruskal-Wallis test for abnormally distributed data. The numerical value was represented by Mean ± SD.
