# Supplementary material for: Completion rates and myelosuppression degrees of cancer patients receiving radiotherapy or chemoradiotherapy unchanged regardless of delay duration after Omicron infection
Source: Sci Rep. 2024 Jun 20;14:14226. doi: 10.1038/s41598-024-65019-y (PMC11190149; doi:10.1038/s41598-024-65019-y)
Supplement: Supplementary file 2 — Supplementary Table S2. [file 41598_2024_65019_MOESM2_ESM.pdf]

Supplementary Table S2. Clinical characteristics of tumor patients who did not complete treatment

Clinical characteristics	Non-COVID-19 group (n=14)	<10-d COVID-19 group (n=13)	≥10-d COVID-19 group (n=15)
Cancer type			
Lung cancer	9	7	12
Head and neck cancer	3	3	2
Brain cancer	1		
Esophageal cancer	1	1	
Cholangiocarcinoma		1	
Gastric cancer		1	1
Radiotherapy position			
Lung	6	3	3
Head and neck	2	2	2
Brain	1	3	3
Esophageal and Chest	2	1	1
Bone	3	1	5
Bile duct		1	
Liver		2	
Adrenal gland			1
