# Supplementary material for: Completion rates and myelosuppression degrees of cancer patients receiving radiotherapy or chemoradiotherapy unchanged regardless of delay duration after Omicron infection
Source: Sci Rep. 2024 Jun 20;14:14226. doi: 10.1038/s41598-024-65019-y (PMC11190149; doi:10.1038/s41598-024-65019-y)
Supplement: Supplementary file 3 — Supplementary Table S3. [file 41598_2024_65019_MOESM3_ESM.pdf]

Supplementary Table S3. Reasons of tumor patients who did not complete treatment

Events	Non-COVID-19 group (n=14)	<10-d COVID-19 group (n=13)	≥10-d COVID-19 group (n=15)
Pneumonia caused by COVID-19		4	3
Pneumonia caused by MDRO	4		1
Common pneumonia	3		
Radiation Pneumonitis	1		
Myelosuppression	5	2	1
Active withdrawal of treatment	1	3	4
Radiation induced oral mucositis			2
Esophageal fistula			1
Deep vein thromboses			1
Gastrointestinal reactions		1	1
Abnormal liver function		1	1
Electrolyte imbalance		1	
Headache		1	

MDRO: Multi-drug Resistant Organism.
